# Phylogeny of the Infraorder Pentatomomorpha Based on Fossil and Extant Morphology, with Description of a New Fossil Family from China

**DOI:** 10.1371/journal.pone.0037289

**Published:** 2012-05-24

**Authors:** Yunzhi Yao, Dong Ren, David A. Rider, Wanzhi Cai

**Affiliations:** 1 Key Laboratory of Insect Evolution and Environmental Changes, Capital Normal University, Beijing, China; 2 Department of Entomology, North Dakota State University, Fargo, North Dakota, United States of America; 3 Department of Entomology, China Agricultural University, Yuanmingyuan West Road, Beijing, China; 4 State Key Laboratory of Palaeobiology and Stratigraphy, Nanjing Institute of Geology and Palaeontology, CAS, Nanjing, China; University of Akron, United States of America

## Abstract

**Background:**

An extinct new family of Pentatomomorpha, Venicoridae Yao, Ren & Cai **fam. nov.**, with 2 new genera and 2 new species (*Venicoris solaris* Yao, Ren & Rider **gen. & sp. nov.** and *Clavaticoris zhengi* Yao, Ren & Cai **gen. & sp. nov.**) are described from the Early Cretaceous Yixian Formation in Northeast China.

**Methodology/Principal Findings:**

A cladistic analysis based on a combination of fossil and extant morphological characters clarified the phylogenetic status of the new family and has allowed the reconstruction of intersuperfamily and interfamily relationships within the Infraorder Pentatomomorpha. The fossil record and diversity of Pentatomomorpha during the Mesozoic is discussed.

**Conclusions/Significance:**

Pentatomomorpha is a monophyletic group; Aradoidea and the Trichophora are sister groups; these fossils belong to new family, treated as the sister group of remainder of Trichophora; Pentatomoidea is a monophyletic group; Piesmatidae should be separated as a superfamily, Piesmatoidea. Origin time of Pentatomomorpha should be tracked back to the Middle or Early Triassic.

## Introduction

The Pentatomomorpha is one of the most diverse groups in the Heteroptera with nearly 15,000 extant species having been recorded worldwide [1]. All of these bugs are terrestrial, mostly plant-feeding (some feed on fungi (Aradoidea), some are predaceous (Pentatomidae, Asopinae)). Many are economically important in agriculture and forestry as a pest of crops. [2], [3]. The phylogenetic relationships among members of the group, however, are not well understood [4]. Schuh and Wheeler *et al.* proposed that the Heteroptera could be divided into seven infraorders (Enicocephalomorpha, Dipsocoromorpha, Gerromorpha, Nepomorpha, Leptopodomorpha, Cimicomorpha and Pentatomomorpha) [5], [6]. The Pentatomomorpha was considered the most advanced with the Cimicomorpha hypothesized as their sister group. The Pentatomomorpha consists of the Trichophora and the Aradoidea. Based on morphological evidence, Schaefer argued that the Aradoidea may be either the sister group of the Trichophora or the Cimicomorpha+Pentatomomorpha [7]. Following, the Aradoidea was raised to infraorder rank, Aradomorpha, by Sweet [8]. But this view has not been recognized by most scholars. Within the Trichophora, only the Pentatomoidea is consistently recognized as monophyletic. The relationship among the superfamilies of the remaining Trichophora is very uncertain. Henry & Froeschner and Li & Zheng accepted 5 superfamilies (Coreoidea, Pyrrhocoroidea, Idiostoloidea, Lygaeoidea, and Piesmatoidea) [9], [10]. Schuh & Slater favored 3 groups, including the Coreoidea, Pyrrhocoroidea and Lygaeoidea [1]. Based on morphological characters, Henry recognized 4 superfamilies (Coreoidea, Pyrrhocoroidea, Idiostoloidea and Lygaeoidea) [11]. More recently, molecular and morphological evidence have been studied in combination. Li *et al.* recognized the remainder of Trichophora as a paraphyletic group based on nuclear 18 S rDNA and mitochondrial DNA sequences [12]; Xie *et al.* performed Bayesian analysis with the 18 S rRNA data, and reported the hypothesis of (Pentatomoidea + (Pyrrhocoroidea + (Coreoidea + Lygaeoidea))) [13]; Hua *et al.* [14] used the mitochondrial genomes of Pentatomomorpha and obtained the same results as Xie *et al.* [13]. The results of these studies indicate that it may be difficult to determine phylogenetic relationships based on extant material; the use of fossil data may provide additional needed information.

Fossil Pentatomomorpha have been studied for over one hundred years, but thus far, there has been only one attempt to combine fossil and extant morphological characters to clarify the phylogenetic relationships within Pentatomomorpha [15]. Reasons for this are two-fold: first, it is very difficult to obtain fossil specimens of true bugs, many fossil species are represented by a single specimen, or sometimes even based on fragmentary remnants such as isolated hemelytra [Bibr pone.0037289-Handlirsch1] [Bibr pone.0037289-BeckerMigdisova1] [Bibr pone.0037289-Popov1] [16−19]; second, Mesozoic fossils of the infraorder Pentatomomorpha are rare with only 7 families (plus the extinct family Pachymeridiidae) and 35 species known (Table S1) and nearly 40 species (their family affinity uncertain) apparently belonging to the Pentatomomorpha (Table S3).

**Figure 1:**
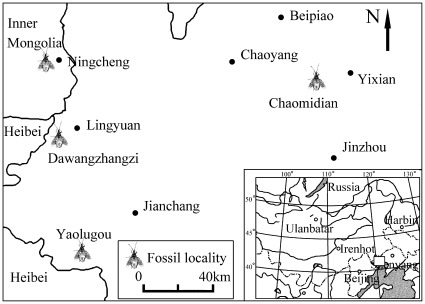
Map of China with the fossil localities indicated indication of fossil locality.

Fortunately, in recent years, our laboratory has collected hundreds of specimens of Pentatomomorpha: from the Jiufotang Formation, in Luzhougou, Yaolugou Town, Jianchang County, Huludao City, Liaoning Province; Yixian Formation, in Chaomidian Village, Beipiao City, Liaoning Province; and the Jiulongshan Formation, in Daohugou Village, Shantou Town, Ningcheng County, Inner Mongolia Autonomous Region, China (Figure 1). Among these specimens are many complete specimens with not only large characters (e.g., antennae, rostrum, wings, legs, abdomen and ovipositor), but also many important small characters preserved for classification (e.g., ocelli, sent glands and spiracles) [Bibr pone.0037289-Yao1] [Bibr pone.0037289-Yao2] [Bibr pone.0037289-Yao3] [Bibr pone.0037289-Yao4] [20−24]. These specimens have been very useful in inferring phylogenetic relationships and ancient evolutionary events of the Pentatomomorpha.

Recently, we recovered over two hundred well-preserved specimens, including 36 parts and counterparts, from the ‘Jianshangou Bed’ in the lower part of the Yixian Formation at Chaomidian Village near the town of Shangyuan, 28 km southeast of Beipiao City, Liaoning Province, China. Based upon careful examination of these well-preserved specimens, we herein erect a new family, Venicoridae Yao, Ren & Cai **fam. nov.**, with two new genera and species. These new fossils provide new evidence for studying the origin and evolution of the Pentatomomorpha. The Yixian Formation, which is considered an important component part of the Jehol entomofauna of north China, contains a diverse insect fauna composed of complete specimens of Ephemeroptera, Odonata, Plecoptera, Blattodea, Orthoptera, Hemiptera, Neuroptera, Coleoptera, Hymenoptera and Diptera [Bibr pone.0037289-Hong1] [Bibr pone.0037289-Ren1] [Bibr pone.0037289-Ren2] [Bibr pone.0037289-Liu1] [Bibr pone.0037289-Yao6] [25−30]. The age of the Yixian Formation is considered to be the Early Cretaceous (125Ma) [Bibr pone.0037289-Luo1] [Bibr pone.0037289-Chen1] [31−33].

**Figure 2:**
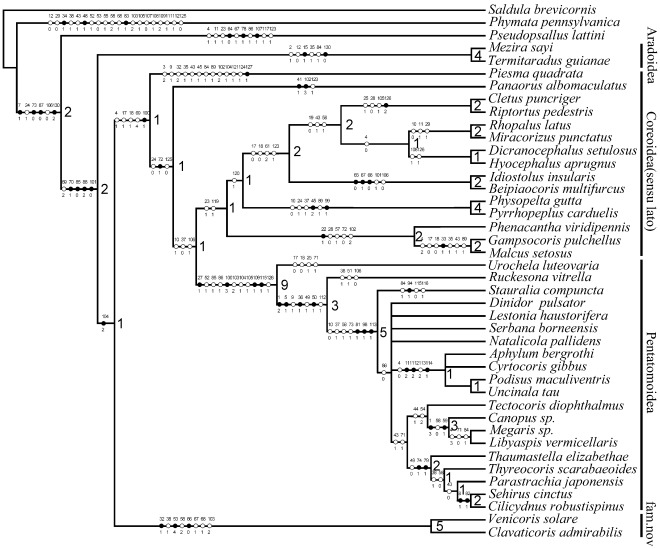
The strict consensus tree of 15 most parsimonious trees with Bremer support values (near branch nodes). (•) Non-homoplasious; (○) homoplasious.

## Results

Major conclusions of the phylogenetic analysis include: Pentatomomorpha is a monophyletic group; Aradoidea and the Trichophora are sister groups; the fossil specimens analyzed form a new family, Venicoridae, and are treated as the sister group of remainder Trichophora; and Pentatomoidea is a monophyletic group. The view that the remainder of Trichophora (except Venicoridae fam. nov) should be assigned to 6 superfamilies (Pentatomoidea, Coreoidea, Pyrrhocoroidea, Idiostoloidea, Lygaeoidea, and Piesmatoidea) and that Piesmatidae is actually a specialized group that should be separated as a superfamily, Piesmatoidea, was supported by our study. Origin time of Pentatomomorpha should be earlier than the existing fossil record, and may be tracked back to the Middle or Early Triassic.

**Figure 3:**
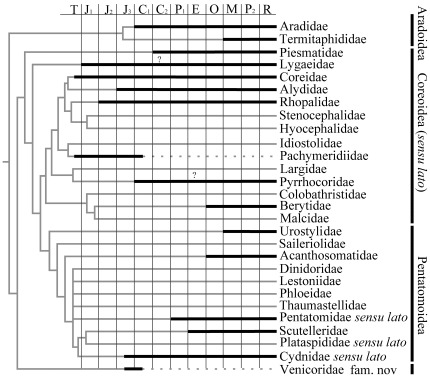
Inferred cladogram, arranged to reflect occurrences of the families in the fossil record. Stratigraphic ranges are based on the oldest confirmed occurrence in each family. Ghost ranges are indicated as gray lines.

## Materials and Methods

### Nomenclatural Acts

The electronic version of this document does not represent a published work according to the International Code of Zoological Nomenclature (ICZN), and hence the nomenclatural acts contained in the electronic version are not available under that Code from the electronic edition. Therefore, a separate edition of this document was produced by a method that assures numerous identical and durable copies, and those copies were simultaneously obtainable (from the publication date noted on the first page of this article) for the purpose of providing a public and permanent scientific record, in accordance with Article 8.1 of the Code. The separate print-only edition is available on request from PLoS by sending a request to PLoS ONE, 1160 Battery Street, Suite 100, San Francisco, CA 94111, USA along with a check for $10 (to cover printing and postage) payable to "Public Library of Science".

In addition, this published work and the nomenclatural acts it contains have been registered in ZooBank , the proposed online registration system for the ICZN. The ZooBank LSIDs (Life Science Identifiers) can be resolved and the associated information viewed through any standard web browser by appending the LSID to the prefix "http://zoobank.org/". The LSID for this publication is: urn:lsid:zoobank.org:pub:E0AA4192-8956-4441-A2D6-349AF0B3C5B0.

**Figure 4:**
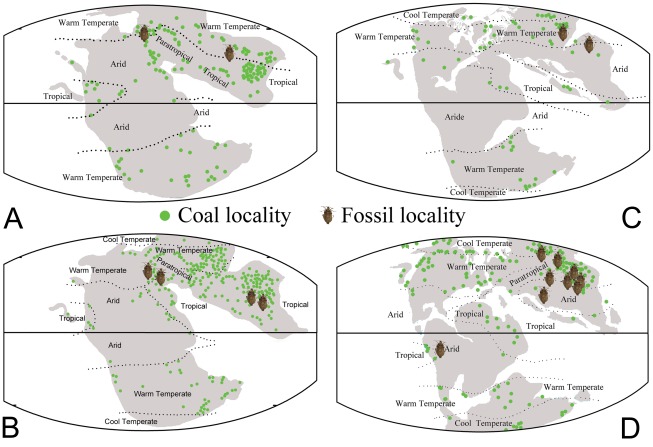
Fossil insect bearing localities. Marked on the Mesozoic palaeocontinental maps of various ages, including pertinent coal localities and climate boundaries (modified from Scotese 2009) [48]. A. Late Triassic (about 210 Ma); B. Early Jurassic (about 195 Ma); C. Late Jurassic (about 152 Ma); D. Early Cretaceous (about 120 Ma).

### Examined Taxa and Terminology

The analyses were based upon the examination of all fossil material deposited in the the Key Laboratory of Insect Evolution and Environmental Changes, College of Life Science, Capital Normal University, Beijing, China. All extant specimens examined were obtained from the Entomological Museum of the North Dakota Insect Reference Collection, Fargo, USA. If actual material was unavailable, the descriptive information for taxa was obtained from correlative literature. All drawings were made using a camera lucida and binocular microscope. The photographs were taken by Nikon DMX1200C and processed using Adobe Photoshop CS2. Morphological terminology in this paper mainly follows Schuh & Slater [1]. We follow the wing venation nomenclature of Leston [34], and Wootton & Betts [35], amended by Schuh & Slater [1]. All measurements are in millimeters.

**Figure 5:**
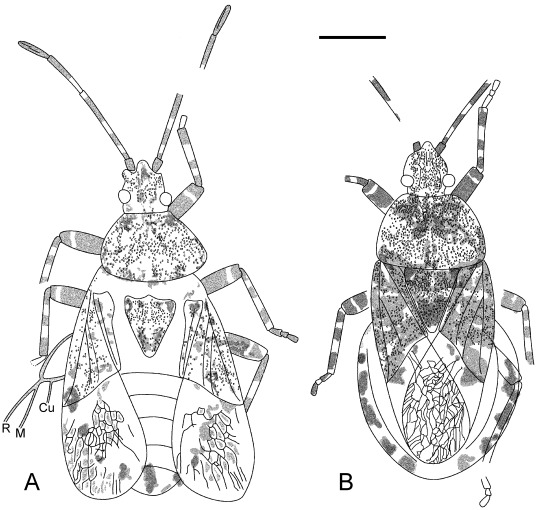
Outline of *Venicoris solaris* Yao, Ren & Rider gen. & sp. nov. A. Holotype, male, CNU-HE-LB2006526; B. paratype, female, CNU-HE-LB2006532. Scale bar  =  2 mm.

**Figure 6:**
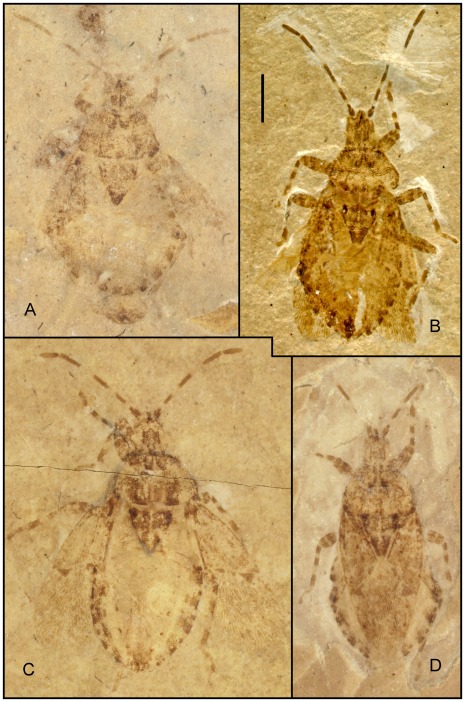
Photographs of *Venicoris solaris* Yao, Ren & Rider gen. & sp. nov. A. Paratype, male, CNU-HE-LB2006513C; B. holotype, male, CNU-HE-LB2006526; C. paratype, female, CNU-HE-LB2006512; D. paratype, female, CNU-HE-LB2006532. (A, C, D under ethanol; B dry).Scale bar  =  2 mm.

**Figure 7:**
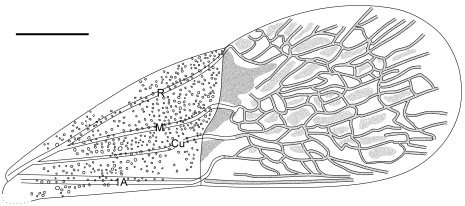
Outline of forewing of *Venicoris solaris* Yao, Ren & Rider gen. & sp. nov. paratype, female, CNU-HE-LB2006614. Scale bar  =  1 mm.

**Figure 8:**
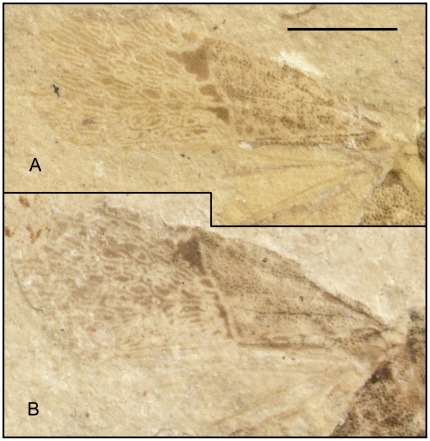
Photographs (dry) of forewing of *Venicoris solaris* Yao, Ren & Rider gen. & sp. nov. paratype. A. female, CNU-HE-LB2006614; B. female, CNU-HE-LB2006624C. Scale bar  =  2 mm.

**Figure 9:**
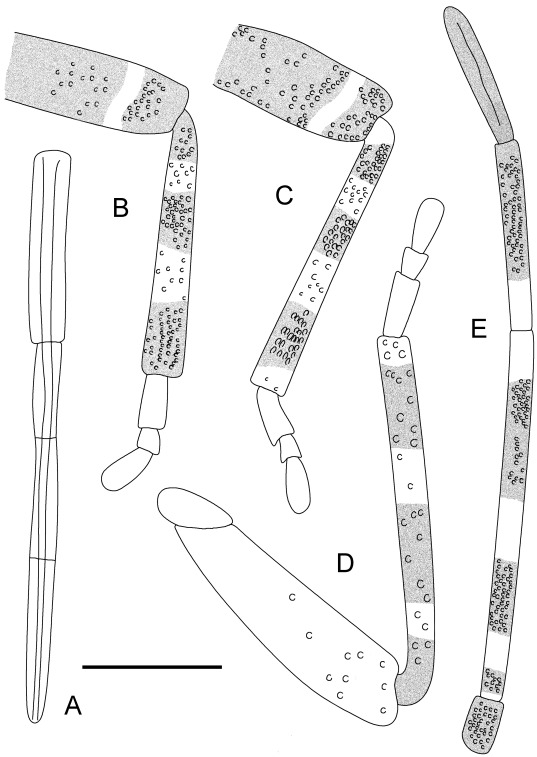
Outline of *Venicoris solaris* Yao, Ren & Rider gen. & sp. nov. A. Rostrum, paratype, female, CNU-HE-LB2006643; B. fore leg, paratype, male, CNU-HE-LB2006523; C. middle leg, holotype, male, CNU-HE-LB2006526; D. hind leg, paratype, male, CNU-HE-LB2006524; E. antenna, holotype, male, CNU-HE-LB2006526. Scale bar  =  1 mm.

**Figure 10:**
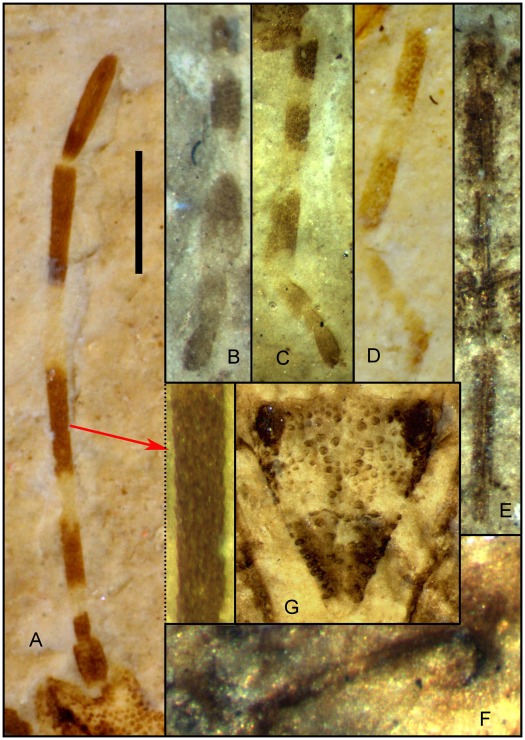
Photograph (under ethanol) of *Venicoris solaris* Yao, Ren & Rider gen. & sp. nov. A. Antenna, holotype, male, CNU-HE-LB2006526; B. Apex of second antennal segment, holotype, male, CNU-HE-LB2006526; C. foreleg, paratype, male, CNU-HE-LB2006512; D. middle leg, holotype, male, CNU-HE-LB2006526; E. hind leg, paratype, male, CNU-HE-LB2006539; F. rostrum, paratype, female, CNU-HE-LB2006643; G. scent-gland, paratype, female, CNU-HE-LB2006627; H. scutellum, paratype, male, CNU-HE-LB2006550. Scale bar  =  1 mm for A, C−F, H; 0.4 mm for B; 0.3 mm for G.

**Figure 11:**
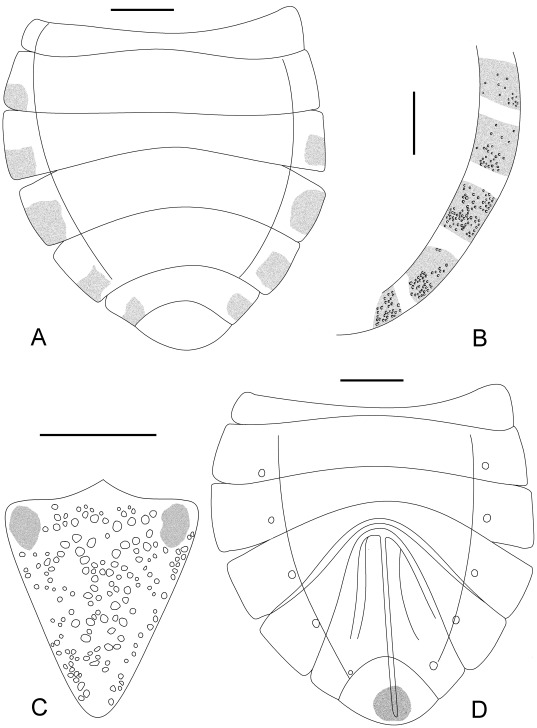
Outline of *Venicoris solaris* Yao, Ren & Rider gen. & sp. nov. A. Dorsal view of female abdomen, paratype, female, CNU-HE-LB2006585; B. connexivum, paratype, female, CNU-HE-LB2006606; C. scutellum, paratype, male, CNU-HE-LB2006550; D. ventral view of female abdomen and ovipositor, paratype, female, CNU-HE-LB2006585. Scale bar  =  1 mm.

**Figure 12:**
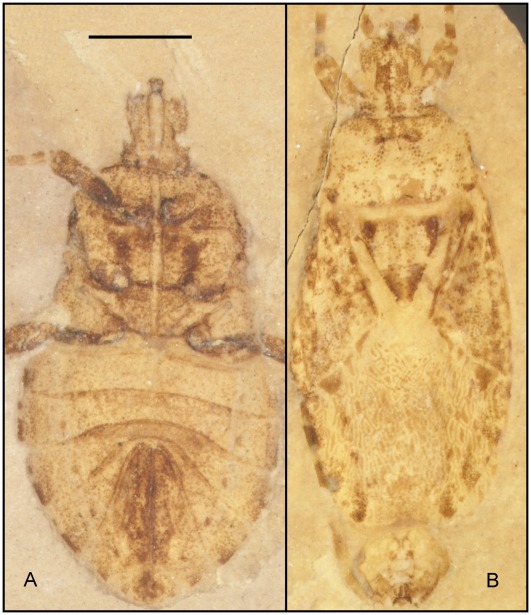
*Venicoris solaris* Yao, Ren & Rider gen. & sp. nov., photograph (under ethanol). A. paratype, female, CNU-HE-LB2006585; B. paratype, male, CNU-HE-LB2006550. Scale bar  =  1 mm.

### Cladistic Analysis

The out-groups were chosen from representatives of the infraorder Leptopodomorpha (Saldidae: *Saldula brevicornis* Rimes) and Cimicomorpha (Reduviidae: *Phymata pennsylvanica* Handlirsch, and Miridae: *Pseudopsallus lattini* Stonedahl and Schwartz). The Leptopodomorpha has traditionally been regarded as a more basal group than the Pentatomomorpha and the Cimicomorpha within the Heteroptera, and the Cimicomorpha has been regarded as a sister group of the Pentatomomorpha [6], [10], [36]. The 39 ingroup terminal taxa include five fossil taxa and 34 extant taxa. A complete list of the taxa used in the phylogenetic analysis is given in Table S2.

There were 130 characters used in the analysis (Text S1). Of these, 93 characters were coded as binary and 37 as multistate. Multistate characters were treated as non-additive. The missing data were scored as unknown. The data matrix is provided in Table S2. Analysis of the character matrix was performed on NONA version 2.0 [37] with the WinClada version 1.00.08 interface [38]. Runs were conducted using the following commands: Maximum trees to keep (hold)  =  10000; Number of replications (mult*N)  =  10, and the multiple TBR+TBR search strategy. Bremer support values were obtained using command files composed by TreeRot [39] in conjunction with the heuristic search algorithm in PAUP v4.0b10 [40].

**Figure 13:**
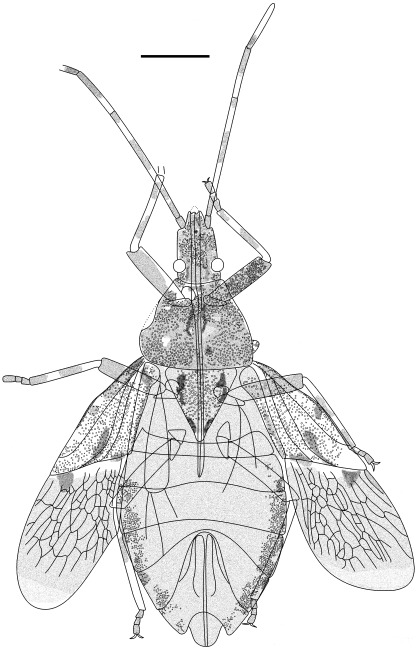
Outline of *Clavaticoris zhengi* Yao, Ren & Cai gen. & sp. nov. Holotype, female, CNU-HE-LB2009016PC. Scale bar  =  2 mm.

**Figure 14:**
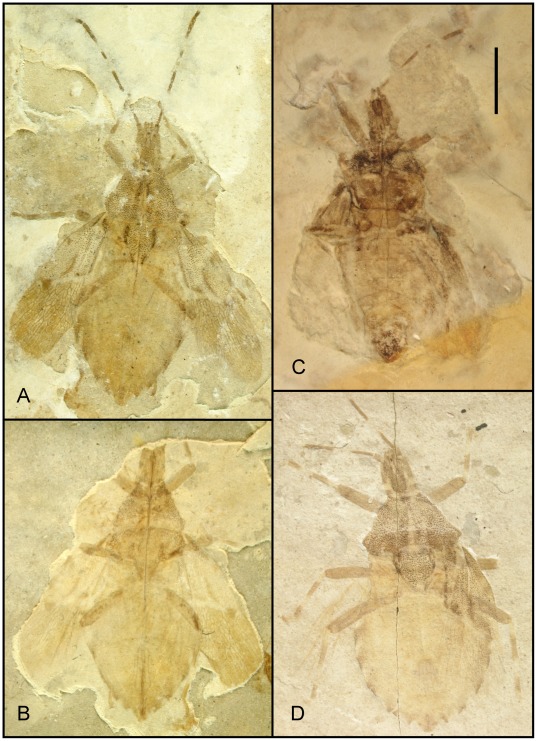
Photographs of *Clavaticoris zhengi* Yao, Ren & Cai gen. & sp. nov. A, B. Holotype, female, CNU-HE-LB2009016PC; C. paratype, male, CNU-HE-LB2012088; D. paratype, female, CNU-HE-LB2012087C. (A, B, D dry; C under ethanol). Scale bar  =  4 mm.

## Discussion

### Phylogenetic Analysis

Analysis of the character matrix yielded 15 most parsimonious trees (Length  =  424, consistency (CI)  =  0.42, retention index (RI)  =  0.69). The strict consensus cladogram of all MP trees is shown in Figure 2, with unambiguous characters mapped. Major conclusions of the phylogenetic analysis include: Pentatomomorpha is a monophyletic group; Aradoidea and the Trichophora are sister groups; the fossils discussed in this paper represent a new family, and are treated as the sister group of the remaining Trichophora; Piesmatidae and Lygaeidae are located at the base of the remaining Trichophora; and Coreoidea (*sensu lato*), excluding Piesmatidae and Lygaeidae, are sister to Pentatomoidea. This result is simplified in following ways: Pentatomomorpha =  Aradoidea + (Venicoridae Yao, Ren & Cai **fam. nov.** + (Piesmatidae + (remaining Coreoidea (*sensu lato*) + Pentatomoidea))).

#### Aradoidea

The monophyly of the Pentatomomorpha was well supported by a series of synapomophic characters, such as the distal sector of R+M in hindwing branching (character 70∶1), claws cylindrical (character 85∶0), dorsal arolium absent (character 88∶2) and connexivum on segment VII present (character 101∶0). Our results reaffirm Pentatomomorpha is a monophyletic group as indicated in previous studies. The point of view that the Aradoidea is the sister group of all Trichophora and is located in a basal position in the Pentatomomorpha has also been confirmed. The point of Schaefer [7] and Sweet [8] that the Aradoidea may be either the sister group of Trichophora or Cimicomorpha+Pentatomomorpha and that the Aradidae should be treated as a separate infraorder, Aradomorpha, is not supported by our analysis.

#### Venicoridae Yao, Ren & Cai fam. Nov

According to the present result, the monophyly of the new family is well supported by a series of characters: antennal segment 2 very long, distinctly longer than antennal segment 3, subequal to segments 3 and 4 combined (character 32∶1); pronotum without distinct callus (character 38∶1); claval apices close together but not contiguous, not concealed by scutellum (character 53∶4); connexivum completely exposed (character 58∶2); R, M and Cu veins inosculated at basal of corium (character 66∶0); Sc absent, R and M veins diverging at basal of corium only 1A veins on clavus (character 68∶1); and spiracles on segment VIII in males absent (character 103∶2). Among these, characters 32, 53 and 66 are synapomophic, being shared only by members of the new family. This taxon appears to be the sister group of all remaining Trichophora.

Venicoridae may represent a new superfamily, and may be ancestral to the Trichophora. They have some interestingly characters. For example, the antenna is 4-segmented, with the pedicel very long, subequal to segments 3 and 4 combined (antenna 5-segmented with pedicel subdivided is a characteristic feature of the Pentatomoidea). We regard the long, undivided pedicel as evidence that the 5-segmented antennae evolved from 4-segmented antennae); clavus clubbed, apices close together but not contiguous, not concealed by scutellum, (clavus clubbed and claval commissure lacking, are common with the Pentatomoidea, but usually the apex of clavus is concealed by the scutellum; the scutellum is small and triangular and a distinct claval commissure is common in Coreoidea *sensu lato*). We believe if is premature to describe a new superfamily based only upon the two genera and species described here. In these fossils, there are many informative small characters and in particular some characters in the genitalia, that did not preserve well. More evidence is needed to establish the superfamily.

#### Coreoidea (*sensu lato*)

Since Drake and Davis [41] transferred the Piesmatidae to the Pentatomomorpha, the systematic position of the Piesmatidae within that infraorder has been is debated. The Piesmatidae has been placed within the Lygaeoidea [1], [7], [11]. Others have proposed that the Piesmatidae constituted a separate superfamily, the Piesmatoidea [10], . More recently, Xie *et al.* [13] based on a Bayesian analysis of 18 S rDNA data placed the Piesmatidae within the Lygaeoidea. Li *et al.* [12] came the same conclusion using partial sequences of the *CO*1 and 18 S rDNA. Our analysis supports the basal treatment of the Piesmatidae within the Trichophora (except for the new family, which is basalmost). The Piesmatoidea is supported by a series of characters, including large punctures on the surface of the body, only the metathoracic scent-gland peritreme reticulate (character 45∶1), tarsi two-segmented (character 84∶1), dorsal laterotergites fused with mediotergites (character 89∶1), abdominal spiracles dorsal (character 102∶2), one prespiracular trichobothrium present on sternum 5 and one on 6 (character 104∶1), dorsal abdominal scent gland between segments 4/5 in nymphs absent (character 124∶1), and eclosion fractures hexagonal (character 127∶1).

The status of Lygaeidae is not resolved in our study. Its monophyly is supported by the narrow impressed line or transverse groove present across each callus (character 41∶1), all spiracles dorsal (character 102∶3) and the dorsal abdominal scent gland between segments 3 / 4 in nymphs absent (character 123∶1). However, the proposed autapomorphies for this family are either variable within the family, or not unique to the family. Characters 41 and 102 are also lacking in the other subfamilies within Lygaeidae (except Ischnorhychinae, Lygaeinae and Orsillinae), while character 123 codes similarly for the superfamilies Coreoidea and Idiostoloidea [11]. More taxa need to be sampled to resolve these problems.

The monophyletic subclade of Colobathristidae + Berytidae + Malcidae was supported by the presence of strongly developed sutures between the ocelli and the compound eyes (character 22∶1); antenniferous tubercles arising below the level of the eye, partially obscured by mandibular plates (character 28∶0); the forewing narrowed, corial margin concave, with the base of the abdomen constricted (character 57∶1); hamus on hindwing absent (character 72∶0); and the position of spiracles II, III, and IV dorsal (character 102∶2).

The monophyletic subclade of Pyrrhocoroidea was supported by the presence of long and narrow bucculae, extending half the length of the head or more (character 10∶0); ocelli absent (character 24∶1); pronotum without a distinct collar (character 37∶1); metathoracic scent-gland absent (character 45∶2); dorsal laterotergites fused with mediotergites (character 89∶1); and sutures 4 and 5 incomplete (character 99∶1).

Idiostolidae and the fossil family Pachymeridiidae formed a monophyletic group by having the C vein present (character 65∶0), the Sc, R, and M veins on forewing diverging from a single point (character 67∶1), 1A and 2A present (character 68∶0), connexivum on segment VII absent (character 101∶1), and sternite VII divided by ovipositor (character 106∶0).

The monophyletic subclade of Coreoidea was supported by three homoplasious characters (character 19∶0, character 43∶1, and character 58∶1), but in 15 MP trees its topology does not transfer. Despite the lack of strong support based on the characters chosen for this study, we believe the superfamily Coreoidea is a monophyletic group.

#### Pentatomoidea

Our study supports the monophyly of the Pentatomoidea on the basis of the following characters: antenna with five segments (character 27∶1), scutellum reaching or surpassing an imaginary transverse line crossing the connexivum at apical angles of abdominal segment III (character 52∶1), claws flattened, tapering from base to apex (character 85∶1) and with bristles (character 86∶1), abdominal sterna 2−7 fused sterna (character 100∶3), spiracles on segment VIII in males absent (character 103∶2), paired abdominal trichobothria on sterna 3−7 (character 104∶1), ovipositor platelike (character 105∶1), tergite IX in females not visible dorsally, covered by apically positioned tergite VIII (character 109∶1), sternite VIII in males concealed by segment VII (character 115∶1), and dorsal abdominal scent gland in nymphs double, separated (character 126∶1).

Our study supports of the view of Carver & Woodward [44] and Grazia *et al.* [45] that the Urostylidae is the basal group within the Pentatomoidea. The monophyly of the remaining Pentatomoidea (except Urostylidae) is supported by body ovoid, scarcely longer than wide (character 1∶2), with the head dorso-ventrally flattened, laterally carinate (character 5∶1), mandibular plates enlarged, extending to apex of clypeus (character 9∶1), pronotum hexagonal, semicircular or other (character 36∶1), posterior and humeral angles developed (character 49∶1), scutellum large, longer than one-third length of hemelytron (character 50∶1), and gonapophyses 9 moderately sclerotized to membranous, with second rami thinly sclerotized or obsolete (character 112∶1). In the present study, Saileriolidae is the sister group to the subclade (containing the remainder of Pentatomoidea, not including Urostylidae and Saileriolidae). The monophyletic subclade of the remainding Pentatomoidea exclusive of the Urostylidae and Saileriolidae is well supported by characters that include: a long, narrow bucculae, extending half length of head or more pronotum without collar (character 37∶1), connexivum partially covered by forewing (character 58∶1), coxae of all three pairs of legs equally distant from each other (character 73∶1), fore tibial apparatus present (character 81∶1), sternite II at middle concealed by metasternum (character 98∶1), and gonangulum partially sclerotized or membranous (character 113∶1). The Acanthosomatidae and Pentatomidae *sensu lato* (including Aphylidae and Cyrtocoridae) were found to be monophyletic, a hypothesis concordant with the work of Grazia *et al.* [45].

The monophyletic subclade of Cydnidae *sensu lato* (including Thaumastellidae, Corimelaenidae and Parastrachiidae) was well supported by the posterior and humeral angles of pronotum not developed (character 49∶0), coxae with fringes of setae, bristles or scales (character 74∶1), and fore tibiae with a row of stout setae on lateral margin (character 79∶1). Canopidae, Megarididae and Plataspididae were grouped by the possession of a spheroid body, almost as long as wide (character 1∶3), connexivum completely covered by the scutellum (character 58∶0), and forewing much longer than abdomen, elbowed between membrane and corium, and folded below scutellum in repose (character 59∶1). Scutelleridae is the sister group of the subclade comprising Canopidae, Megarididae and Plataspididae.

### The Fossil Record and Diversity During the Mesozoic

At least 200 genera known in the fossil record within the Pentatomomorpha are assigned to the 16 families. There are fossil representatives from each of the following superfamilies Aradoidea, Piesmatoidea, Lygaeoidea, Coreoidea, Idiostoloidea, Pyrrhocoridae and Pentatomoidea. The geologic ranges of the Pentatomomorpha extend into the Late Triassic, although fossil representatives are only known for Coreidae (Coreoidea) and Pachymeridiidae (Idiostoloidea) [44], [45] during the Late Triassic (Table S1 and Figure 3). These specimens were found in Europe (Late Rhaetian, England) [Bibr pone.0037289-Giebel1] [Bibr pone.0037289-Popov3] [47−49] and China (Toksun conty, Xinjiang Uygur Autonomous Region) [46] (Table S1; Text S1; Figure 4A). The occurrence of these families suggest that the time of origin of the Pentatomomorpha was no later than Late Triassic.

The Pangea Mega-monsoon was in full swing during the Early and Middle Jurassic. The interior of Pangea was very arid and hot; deserts covered what is now the Amazon and Congo rainforests, and since China was surrounded by humid winds, it was lush and verdant [50]. During this period, fossil Pentatomomorpha occurred in Europe (Germany and England) and China (Anhui, Hebei, Liaoning, Hunan province, Guangxi Zhuang Autonomous Region and Inner Mongolia Autonomous Region) (Figure 4B). To date, from the Late Triassic to Late Jurassic the fossils that have been found belong in the Coreoidea (Coreidae, Rhopalidae) [20], [21], [25], Lygaeoidea (Lygaeidae, with family attribution questionable, but apparently belonging to the Pentatomomorpha) [51], [52], and Idiostoloidea (Pachymeridiidae) [16], [22], [24]. This suggests that the Coreoidea *sensu lato* had diverged by the Early Jurassic, although some of the other families have ghost ranges (Figure 3). In addition, they also imply that the Coreoidea *sensu lato* is the oldest branch in the Pentatomomorpha.

From the Middle Jurassic, the supercontinent of Pangea began to break apart. In the Late Jurassic the Central Atlantic Ocean was a narrow ocean separating Africa from eastern North America, and Eastern Gondwana had begun to separate form Western Gondwana. During the Late Jurassic the global climate began to change due to the breakup of Pangea [48]. From the Middle Jurassic to Early Cretaceous, the climate in Europe had dramatically changed, the large area of forest became reduced. But at the same time, the climate of the entire north-eastern part of Eurasia regional became warm and humid, and dense vegetation (Figure 4C, D), suitable for true bugs appeared. The Late Jurassic to Early Cretaceous was an important period for the evolution of pentatomomorphan bugs. During this period, representatives of all superfamilies emerged. The oldest representative of Aradidae occurred in the Early Cretaceous, with records scattered from West Mongolia [53], Russia [54], and Myanmar [55]. This family is the sister group of all Trichophora, and it is located in a basal position to the Pentatomomorpha, but compared with the fossil records of Coreoidea *sensu lato*, its fossils are much younger. Maybe the Aradidae are under-represented in the fossil record and thus have very long ghost ranges (Figure 3). The oldest fossil of the Pentatomoidea (Cydnidae) was found during the Early Cretaceous also [18], [23], [56]. The Pachymeridiidae was well-established during the Mesozoic and became extinct during the Early Cretaceous [57]. After the Early Cretaceous, the climate of Eastern Asia became arid [58]. This was also the time of the biological great extinction in the Late Cretaceous, these bugs almost “disappeared” in Asia from the Middle Cretaceous to the Miocene.

### Taxonomy

Suborder Heteroptera Latreille, 1810 Infraorder Pentatomomorpha Leston, Pendergrast & Southwood, 1954 Superfamily uncertain **Venicoridae Yao, Ren & Cai fam. nov.** urn:lsid:zoobank.org:act:F00AA10B-753E-4D1D-B04A-DCAC903F86BD.

#### Type genus

*Venicoris* Yao, Ren & Rider gen. nov.

#### Diagnosis

Body of moderate to large size, oval; head pentagonal; antenna 4-segmented, base arising below level of eye, first segment short, not extending beyond head apex, second segment very long; eyes round, ocelli absent; rostrum 4-segmented, straight, appressed to body, more elongated, extending beyond fore coxae; pronotum trapezoidal, without collar, prosternum carinae present; scutellum triangular, claval commissure absent; legs simple, lacking spine, tarsus 3-segmented; forewing macropterous, R, M and Cu inosculated at basal of corium, membrane with reticulate venation, 1A on clavus, clavus tapering, hind wings with a branching distal sector of R+M. Abdomen oval, with wide connexivum, sixth sterna convex, seventh sternum longest, split by ovipositor, ovipositor laciniate, long, subequal in length to half of abdomen, not projecting beyond last paratergites.

#### Distribution

China.

#### *Venicoris* Yao, Ren & Rider gen. nov

urn:lsid:zoobank.org:act:6A856D86-D4C8-4D8E-B17A-9A0416707650.

#### Type species

*Venicoris solaris* Yao, Ren & Rider **sp. nov.**

#### Etymology

The generic name is a combination of the Latin ‘*ven-*’ (‘vein’) and ‘*coris’* (‘bug’). The gender is masculine.

#### Description

Body elongated-oval, about 2 times as long as wide. Antennae, head, pronotum, scutellum, corium, clavus, legs and connexivum densely punctate. Head width and length subequal, shorter than pronotum, clypeus extending beyond mandibular plates, anteocular portion longer than postocular; antennae arising from near apex of head, far from eyes, longer than head and pronotum combined, first segment shortest, second longest and slender, fourth segment fusiform, shorter and stouter than third; rostrum arising from apex of head, extending to mid coxae, first segment thickest and longest, second shortest, fourth longer than third, acute distally; eyes of medium size, projecting over head laterally, interocular space distinctly longer than diameter of eye. Pronotum length shorter than width; scutellum extending to third abdominal segment, length slightly longer than width, basal margin convex, covered under pronotum, apex rounded; scent-gland channel on metathoracic pleuron long, hook-like; coxae and trochanter rounded triangular, femora distinctly thicker than tibiae, fore and mid femora subequal to corresponding tibiae, hind femora shorter than corresponding tibiae, first tarsomere longest, second shortest, third oval. Hemelytron reaching to tip of abdomen, corium venation prominent, Sc absent, R rising from basal part of corium, extending to tip of corium and into membrane, M and Cu rising from basal part of R, parallel, these veins extend into membrane; 1A along inner margin of clavus and ending apex; basal clavus extended. Abdomen oval, with 7 visible segments, ovipositor long, extending through last two abdominal segments.

***Venicoris solaris*** **Yao, Ren & Rider sp. nov.** (Figures 5, 6, 7, 8, 9, 10, 11 and 12) urn:lsid:zoobank.org:act:A9746F63-A468-4CAA-90A3-9B9CA37BE7C6.

#### Description

Length of body 1.8 (male) and 1.7 (female) times body's width. Antenna slender, basal, middle and apical parts of second segment, as well as basal parts of third segment paler, antenna longer than half of body, second segment about 6 times as long as first, third 1.3 times as long as fourth, fourth with a longitudinal ridge; second rostral segment 0.5 times as long as first and 0.8 times as long as third, third about 0.75 times as long as fourth; eyes somewhat prominent, interocular space about 3 times as wide as eye diameter in dorsal view.

Pronotum moderately transverse, 1.6 times as wide as long, anterior margin about 0.5 times as long as posterior, lateral margins convex, anterior and posterior angles weakly rounded; scutellum marginally longer than pronotum at median line, 1.5 times as wide as long; black round markings at basal angles of scutellum, without puncta in markings; femora stout, almost 2 times as thick as corresponding tibiae, femora with pale annulus within apical 1/6 of its length, tibiae with two pale annuli, fore and mid femora subequal to corresponding tibiae in length, tarsi shorter than half length of corresponding tibiae, basal tarsomere over 2 times as long as second and 1.3 times as long as third with third tarsomere oblong-oval; hind legs longer than mid legs, femora 1.2 times as long as tibiae. Base of membrane with three black markings, outer ones largest, middle ones smallest; costal margin of hemelytron weakly convex, anterior margin of corium 0.7 times as long as hemelytron, clavus nearly 6 times as long as wide with length 0.4 times that of hemelytron.

Abdomen broad, about as long as wide, connexivum with black square markings at the posterior angles of each segment, ovipositor one-fourth as long as body.

#### Dimensions (in mm)

Body length 9.3−10.2 (male), 8.3−10.7 (female); maximal width of body 4.3−5.0 (male), 4.6−5.6 (female); length of head 1.5−1.6 (male), 1.8−2.0 (female), width 1.5−1.6 (male), 1.7−1.9 (female); length of antennal segments I−V: 0.3−0.4, 2.3−2.6, 1.3−1.4, 0.9−1.1 (male), 0.3−0.4, 1.9−2.2, 1.1−1.3, 0.8−0.9 (female); length of rostral segments I−IV: 1.4, 0.7, 0.9, 1.2 (male), total 3.5−4.1(female); length of pronotum 2.0−2.2 (male), 1.6−1.9 (female), width 3.1−3.3 (male), 3.3−3.6 (female); length of scutellum 1.8−2.0 (male), 1.5−1.8 (female), width 1.5−1.9 (male), 1.4−1.7 (female); length of hemelytron 6.0−6.7 (male), 5.2−6.0 (female), width 2.0−2.8 (male), 2.1−2.4(female), length of anterior margin corium 3.5−4.0 (male), 3.3−3.6(female), length of clavus 2.2−3.0 (male), 2.1−2.9 (female); width 0.5−0.6 (male), 0.4−0.6 (female); length of fore leg: femur 1.9 (male), 1.9 (female), tibia 1.8−1.9 (male), 1.8−1.9 (female), tarsomeres I−III:0.4, 0.2, 0.4 (male), 0.4, 0.2, 0.3 (female); length of middle leg: femur 1.8−2.0 (male), 2.2(female), tibia 1.8−1.9 (male), 1.9−2.1 (female), tarsomeres I−III:0.3−0.4, 0.1−0.2, 0.3−0.4 (male), 0.4, 0.2, 0.3 (female); length of hind leg: femur 2.3−2.4 (male), 2.5−2.6 (female), tibia 2.6−2.8 (male), 3.4 (female), length of tarsomeres I −III: 0.4−0.5, 0.2, 0.4 (male), 0.5, 0.2, 0.3 (female).

#### Type material

Holotype, male, CNU-HE-LB2006526 (dorsoventrally compressed); paratypes, 84 males: CNU-HE-LB2006523PC,CNU-HE-LB2012076PC (laterally compressed, part and counterpart), CNU-HE-LB2006515−522,CNU-HE-LB2010016,CNU-HE-LB2012077-086 (laterally compressed), CNU-HE-LB2006508PC/509PC/513PC/535PC, CNU-HE-LB2012058PC−060PC (dorsoventrally compressed, part and counterpart), CNU-HE-LB2006524/525, CNU-HE-LB2006527−531/533−568,CNU-HE-LB2012061−075 (dorsoventrally compressed); 125 females: CNU-HE-LB2006532, CNU-HE-LB2006569−610, CNU-HE-LB2012020−057 (dorsoventrally compressed), CNU-HE-LB2006642PC/511PC/626PC/632PC/636PC/638PC, CNU-HE-LB2012009PC−019PC (dorsoventrally compressed, part and counterpart), CNU-HE-LB2006512, CNU-HE-LB2006611−623,CNU-HE-LB2012004−008 (laterally compressed), CNU-HE-LB2006624PC/628PC/630PC/634PC/640PC, CNU-HE-LB2012001PC/002PC/003PC (laterally compressed, part and counterpart).

#### Locality and horizon

Huangbanjigou, Chaomidian Village, Beipiao City, Liaoning Province, China. Late Jurassic to Early Cretaceous, Yixian Formation.

#### Etymology

The name is derived from the Latin solaris (‘sun’). The gender is masculine.

#### *Clavaticoris* Yao, Ren & Cai gen. nov

urn:lsid:zoobank.org:act:B4DC74F1-C8E7-4FC0-B0D4-5606919C0772.

#### Type species

*Clavaticoris zhengi* Yao, Ren & Cai **sp. nov.**

#### Etymology

The generic name is a combination of the Latin ‘*clavat-’*(‘claval’) and ‘*coris’* (‘bug’). The gender is masculine.

#### Description

Body elongated-oval, about 2.5 times as long as wide. Head, pronotum, scutellum, corium, clavus, legs and connexivum densely punctate. Head with clypeus extending beyond mandibular plates, length longer than width, as long as pronotum, anteocular portion longer than postocular; antennae arising from near apex of head, far from eyes, longer than head and pronotum combined, first segment shortest, second longest and slender, third and fourth segment subequal in length; rostrum arising from apex of head, reaching to third sternite of abdomen; eyes of medium size, projecting over head laterally, interocular space distinctly longer than diameter of eye. Pronotum length shorter than width; scutellum extending to second abdominal segment, length as long as width, apex rounded; coxae and trochanter rounded triangular, femora distinctly thicker than tibiae, femora shorter than corresponding tibiae, first and third tarsomere subequal, second shortest. Hemelytron reaching to tip of abdomen, corium venation prominent, Sc absent, R rising from basal part of corium, ending within apical 1/6 of costal margin of corium, M and Cu rising from basal part of R, parallel, basal clavus extended. Abdomen oval, with 7 visible segments, ovipositor long, extending through last two abdominal segments.

Remarks: This new genus is closely related to *Venicoris* Yao, Ren & Rider **gen. nov.**, but easily differs from the latter in that the body is about 2.5 times as long as wide (vs. about 2 times), body length is 18 mm (vs. never over 12 mm), with rostrum reaching to third sternite of abdomen (vs. extending to mid coxae) and R ending within apical 1/6 of costal margins of corium (vs. R extending to tip of corium and into membrane).

*Clavaticoris zhengi* Yao, Ren & Cai **sp. nov.** (Figures 13−14) urn:lsid:zoobank.org:act:C0AE17F8-B8DC-4A6D-B385-0C258D8E05FE.

#### Description

Length of body 2.6 times of body width. Antenna slender, basal, middle and apical parts of second segment, as well as basal parts of third paler; antenna longer than half of body length, second segment 2.4 times as long as third; eyes somewhat prominent, interocular space about 2 times as wide as eye diameter in dorsal view.

Pronotum 1.3 times as wide as long, anterior margin about 0.4 times as long as posterior margin, lateral margins convex, anterior and posterior angles feebly rounded; scutellum weakly shorter than pronotum at median line, width equal to length, with black markings at basal angles and apex of scutellum; femora stout, almost 2 times as thick as corresponding tibiae, tibiae with two pale annuli, tarsi shorter than half of corresponding tibiae, hind legs longer than mid legs, femora 1.3 times as long as tibiae. Base of membrane with one black triangular marking; costal margin of hemelytron weakly convex, anterior margin of corium 0.6 times as long as hemelytron, clavus nearly 6 times as long as wide and 0.4 times as long as hemelytron length.

Abdomen broad, longer than wide, connexivum with black square markings at the posterior angles of each segment, ovipositor one-fourth as long as body.

#### Dimensions (in mm)

Female:body length 18.5; maximal width of body 7.2; length of head 3.3, width 2.1; length of antennal segments I−IV: 0.5, 5.1, 2.1, 2.1; total rostral length 11.4; length of pronotum 3.6, width 4.8; length of scutellum 3.0, width 3.0; length of hemelytron 11.4, width 4.0; length of anterior margin corium 6.2; length of clavus 4.2, basal width 0.8; length of foreleg: femur 2.7, tibia 3.0, tarsomeres I−III: 0.5, 0.3, 0.5; length of middle leg: femur 3.2, tibia 3.5, tarsomeres I−III: 0.3, 0.2, 0.5; length of hind leg: femur 3.6, tibia 4.5, tarsomeres I −III: 0.4, 0.2, 0.4.

#### Type material

Holotype, female,CNU-HE-LB2009016PC (dorsoventrally compressed, part and counterpart), paratypes, 1 male: CNU-HE-LB2012088 (dorsoventrally compressed), 1 female:CNU-HE-LB2012087PC (dorsoventrally compressed, part and counterpart).

#### Locality and horizon

Huangbanjigou, Chaomidian Village, Beipiao City, Liaoning Province, China. Late Jurassic to Early Cretaceous, Yixian Formation.

#### Etymology

This species is named in honor of Dr. Leyi Zheng (Institute of Entomology, College of Life Science, Nankai University, Tianjin, China) for his outstanding contribution to the study of Chinese Heteroptera.

## Supporting Information

Table S1 Geographical and stratigraphic distribution of the known fossil species in Pentatomomorpha from the Mesozoic. (T_3_ = Late Triassic, J_1_ =  Early Jurassic, J_2_ =  Middle Jurassic, J_3_ =  Late Jurassic, K_1_ =  Early Cretaceous; * indicates amber specimens).
(DOC)

Table S2 Character matrix of 130 characters for the 42 taxa included in this study. (*denotes a fossil species).
(DOC)

Table S3 **Geographical and stratigraphic distribution of the questionable known fossil species in Pentatomomorpha from the Mesozoic.** (The following genera and species, apparently belonging to the Pentatomomorpha, are too poorly known to permit assignment to families. Families uncertain. T3 = Late Triassic, J1 =  Early Jurassic, J2 =  Middle Jurassic, J3 =  Late Jurassic, K1 =  Early Cretaceous; all of these specimens are compression fossils).
(DOC)

Text S1 **Character descriptions.**
(DOC)
