# Supplementary material for: Phylogeny of the Infraorder Pentatomomorpha Based on Fossil and Extant Morphology, with Description of a New Fossil Family from China
Source: PLoS One. 2012 May 24;7(5):e37289. doi: 10.1371/journal.pone.0037289 (PMC3360028; doi:10.1371/journal.pone.0037289)
Supplement: Table S1 — Geographical and stratigraphic distribution of the known fossil species in Pentatomomorpha from the Mesozoic. (T3 = Late Triassic, J1 = Early Jurassic, J2 = Middle Jurassic, J3 = Late Jurassic, K1 = Early Cretaceous; * indicates amber specimens). (DOC) [file pone.0037289.s001.doc]

**Table S1**. Geographical and stratigraphic distribution of the known fossil species in Pentatomomorpha from the Mesozoic. (T_3_=Late Triassic, J_1_= Early Jurassic, J_2_= Middle Jurassic, J_3_= Late Jurassic, K_1_= Early Cretaceous; * indicates amber specimens).

| *Family* | *Genus and species* | *Age* | *Formation* | *Locality* | Reference |
| --- | --- | --- | --- | --- | --- |
| Aradidae | *Aradus nicbolasi* | K _1_ |  | West Mongolia/ Siberian | [59] |
|  | *Aradus creticus* | K _1_ | Olskaya | Ten'ki District, Magadan Region, Russia | [54] |
|  | *Archearadus burmensis* | K _1_ |  | Myanmar | [60] |
| Kobdocoridae | *Kobdocoris aradinus* | K _1_ | Gurvan-Eren | West Mongolia | [18] |
| Pachymeridiidae | *Neomeridium* *trifurcum* | J_1_ | Charmouth | Flatstones, Stonebarrow, Charmouth, Dorset, England | [61] |
|  | *Pachymeridium dubium* | J_1_ |  | Dobbertin, Mecklenburg in Germany | [62] |
|  | *Sisyrocoris* *rudis* | J_1_ |  | Dobbertin, Mecklenburg in Germany | [11] |
|  | *Apsicoria semideleta* | J_1_ |  | Dobbertin, Mecklenburg in Germany | [16] |
|  | *Cathalus alutaceus* | J_1_ |  | Dobbertin, Mecklenburg in Germany | [16] |
|  | *Sinopachymeridium* *popovi* | J_2_ | Jiulongshan | Daohugou Village, Shantou Town, Ningcheng County, Inner Mongolia Autonomous Region | [24] |
|  | *Viriosinervis stolidus* | J_2_ | Jiulongshan | Daohugou Village, Shantou Town, Ningcheng County, Inner Mongolia Autonomous Region, China | [24] |
|  | *Niticoris regillus* | J_2_ | Jiulongshan | Daohugou Village, Shantou Town, Ningcheng County, Inner Mongolia Autonomous Region, China | [24] |
|  | *Beipiaocoris multifurcus* | K_1_ | Yixian | Chaomidian Village, Beipiao City, Liaoning Province | [24] |
|  | *Bellicoris mirabilis* | K_1_ | Yixian | Chaomidian Village, Beipiao City, Liaoning Province | [24] |
|  | *Peregrinpachymeridium comitcola* | J_2_ | Jiulongshan | Daohugou Village, Shantou Town, Ningcheng County, Inner Mongolia Autonomous Region, China | [63] |
|  | *Corollpachymeridium heteroneurus* | J_2_ | Jiulongshan | Daohugou Village, Shantou Town, Ningcheng County, Inner Mongolia Autonomous Region, China | [63] |
|  | *Pachycoridium* *letum* | K_1_ | Gurvan-Eren | West Mongolia | [18] |
| Piesmatidae | *Cretopiesma suukyiae** | K_2_ |  | Northern province of Kachin, near Myitkyina, Myanmar | [64] |
| Coreidae | *Kerjiecoris oopsis* | T_3_ | Huangshanjie | Toksun conty, Xinjiang Uygur Autonomous Region, China | [46] |
|  | *Hebeicoris longa* | J_2_ | Jiulongshan | Zhouyingzi village, Luanping County, Hebei Province, China | [25] |
|  | *Hebeicoris luanpingensis* | J_2_ | Jiulongshan | Zhouyingzi village, Luanping County, Hebei Province, China | [25] |
|  | *Sinocoris ovata* | J_2_ | Haifanggou | Beipiao City, Liaoning Province | [25] |
|  | *Hebeicoris xinboensis* | K_1_ | Dabeigou | Weichang County, Hebei Province, China | [65] |
|  | *Weichangicoris daobaliangensis* | K_1_ | Dabeigou | Weichang County, Hebei Province, China | [65] |
|  | *Bibiticen hebeiensis* | K_1_ | Yixian | Chengde City, Hebei Province, China | [65] |
| Alydidae | *Monstrocoreus quadrimaculatus* | J_3_ | Karatau | Karatau-Mikhailovka ,Algabasskii District,, Chimkent Oblast, Kazakhstan | [66] |
| Rhopalidae | *Miracorizus punctatus* | J_2_ | Jiulongshan | Daohugou Village, Shantou Town, Ningcheng County, Inner Mongolia Autonomous Region | [20] |
|  | Longiclavula calvata | J_2_ | Jiulongshan | Daohugou Village, Shantou Town, Ningcheng County, Inner Mongolia Autonomous Region | [20] |
|  | *Originicorizus pyriformis* | J_2_ | Jiulongshan | Daohugou Village, Shantou Town, Ningcheng County, Inner Mongolia Autonomous Region | [21] |
|  | *Quatlocellus liae* | J_2_ | Jiulongshan | Daohugou Village, Shantou Town, Ningcheng County, Inner Mongolia Autonomous Region | [21] |
|  | *Grandicaputus bipunctatus* | J_2_ | Jiulongshan | Daohugou Village, Shantou Town, Ningcheng County, Inner Mongolia Autonomous Region | [21] |
| Cydnidae | *Cilicydnus* *robustispinus* | K_1_ | Yixian | Chaomidian Village, Beipiao City, Liaoning Province | [23] |
|  | *Orienicydnus hongi* | K_1_ | Yixian | Chaomidian Village, Beipiao City, Liaoning Province | [23] |
|  | *Latiscutella santosi* | K _1_ | Codo | Corda City, Maranhão State, Brazil | [56] |
|  | *Pricecoris* *beckerae* | K _1_ | Codo | Corda City, Maranhão State, Brazil | [56] |
|  | *Clavicoris cretaceous* | K _1_ | Gurvan-Eren | West Mongolia | [18] |

Reference

59. Popov YuA (**1989)** New fossil Hemiptera (Heteroptera + Coleorrhyncha) from the Mesozoic of Mongolia. N Jb Geol Paläontol Monatsh 3: 166–181.

60. Heiss E, Grimaldi DA (2002) *Archearadus burmensis* gen. n., sp. n., a remarkable Mesozoic Aradidae in Burmese amber (Heteroptera, Aradidae). Carolinea 59: 99–102.

61. Popov YuA, Dolling WR, Whalley PES (1994) British Upper Triassic and Lower Jurassic Heteroptera and Coleorrhyncha (Insects: Hemiptera). Genus 5: 307–347.

62. Geinitz FE (1880) Der Jura von Dobbertin in Mecklenburg und seine Versteinerunger. Z Dt Geol Ges 32: 510–535.

63. Lu Y, Yao YZ, Ren D (2011) Two new genera and species of fossil true bugs (Heteroptera: Pachymeridiidae) from northeastern China. Zootaxa 2835: 41–52.

64. Grimaldi DA, Engel MS (2008) A Termite Bug in Early Miocene Amber of the Dominican Republic (Hemiptera: Termitaphididae). Am Mus Novit 3619: 1–10.

65. Hong YC (1984) Paleontological atlas of north China Ⅱ Mesozoic Volume. Geological Publishing House, Beijing, China. pp.156–161.

66. Popov YuA (1968) True bugs of the Jurassic Karatau fauna (Heteroptera). In: Rohdendorf BB, Jurassic insects of Karatau, Academy of Sciences of the USSR, Section of General Biology, Publishing House "Nauka", pp.99–113.
