# Supplementary material for: Phylogeny of the Infraorder Pentatomomorpha Based on Fossil and Extant Morphology, with Description of a New Fossil Family from China
Source: PLoS One. 2012 May 24;7(5):e37289. doi: 10.1371/journal.pone.0037289 (PMC3360028; doi:10.1371/journal.pone.0037289)
Supplement: Table S3 — Geographical and stratigraphic distribution of the questionable known fossil species in Pentatomomorpha from the Mesozoic. (The following genera and species, apparently belonging to the Pentatomomorpha, are too poorly known to permit assignment to families. Families uncertain. T3 = Late Triassic, J1 = Early Jurassic, J2 = Middle Jurassic, J3 = Late Jurassic, K1 = Early Cretaceous; all of these specimens are compression fossils). (DOC) [file pone.0037289.s003.doc]

**Table S3**. Geographical and stratigraphic distribution of the questionable known fossil species in Pentatomomorpha from the Mesozoic.（The following genera and species, apparently belonging to the Pentatomomorpha, are too poorly known to permit assignment to families. Families uncertain. T_3_=Late Triassic, J_1_= Early Jurassic, J_2_= Middle Jurassic, J_3_= Late Jurassic, K_1_= Early Cretaceous; all of these specimens are compression fossils）.

| *Previous Family Designation* | *Genera and species* | *Age* | *Formation* | *Locality and Horizon* | Reference |
| --- | --- | --- | --- | --- | --- |
| Pachymeridiidae | *Pachymerus zucholdi* | T_3_ |  | England | [75] |
|  | *Psychrocoris cuneifera* | J_1_ |  | Dobbertin, Mecklenburg in Germany | [76] |
|  | *Hypocimex membranaceus* | J_1_ |  | Dobbertin, Mecklenburg in Germany | [16] |
|  | *Cretacoris scutellinus* | K_1_ | Zaza | eastern Transbaikalia of Siberia | [19] |
|  | *Positocoris sibiricus* | K_1_ | Zaza | eastern Transbaikalia of Siberia | [19] |
|  | *P. angustus* | K_1_ | Zaza | eastern Transbaikalia of Siberia | [19] |
|  | *P. longirostris* | K_1_ | Zaza | eastern Transbaikalia of Siberia | [19] |
|  | *P. minimus* | K_1_ | Zaza | eastern Transbaikalia of Siberia | [19] |
|  | *P. intermedius* | K_1_ | Zaza | eastern Transbaikalia of Siberia | [19] |
|  | *Pronotaphanus minor* | K_1_ | Zaza | eastern Transbaikalia of Siberia | [19] |
|  | *Takshania implicata* | K_1_ | Zaza | eastern Transbaikalia of Siberia | [19] |
|  | *T. modesta* | K_1_ | Zaza | eastern Transbaikalia of Siberia | [19] |
|  | *T. transversalis* | K_1_ | Zaza | eastern Transbaikalia of Siberia | [19] |
|  | *Aphanocoris turgensis* | K_1_ | Zaza | eastern Transbaikalia of Siberia | [19] |
|  | *A. porrectus* | K_1_ | Zaza | eastern Transbaikalia of Siberia | [19] |
|  | *A. femoratus* | K_1_ | Zaza | eastern Transbaikalia of Siberia | [19] |
|  | *A. parallelus* | K_1_ | Zaza | eastern Transbaikalia of Siberia | [19] |
|  | *Corrivalius perbonus* | K_1_ | Zaza | eastern Transbaikalia of Siberia | [19] |
| Coreidae | *Sinocoris oblonga* | J_2_ | Haifanggou | Beipiao City, Liaoning Province | [25] |
|  | *Karatavocoris* *asiatica* | J_3_ | Karatau | Karatau-Mikhailovka ,Algabasskii District,, Chimkent Oblast, Kazakhstan | [17] |
|  | *Corioides fortus* | K_1_ | Shahai | Kazuo County, Liaoning Province, China | [77] |
|  | *Kezuocoris liaoningensis* | K_1_ | Shahai | Kazuo County, Liaoning Province, China | [77] |
| Pyrrhocoridae | *Mesopyrrhocorix fasciata* | K_1_ | Laiyang | Laiyang County, Shandong Province, China | [78] |
| Lygaeidae | *Hunanilarva micra* | J_2_ | Guanyintan | Qiyang county, Hunan Province, China | [52] |
|  | *Leipolygaeus similis* | J_1_ | Hanshan | Pengzhuang village, Hanshan county, AnhuiProvince, China | [51] |
|  | *Cymocoris basalis* | K_1_ | Gurvan-Eren | West Mongolia | [18] |
|  | *Lygaenocoris prynadai* | J_3_ | Karatau | Karatau Range, Algabasskii District, Chimkent Oblast, South Kazakhstan | [79] |
| Protocoridae | *Protocoris indistinctus* | J_1_ | Charmouth | Flatstones, Stonebarrow, Charmouth, Dorset, England | [61] |
|  | *Pallicoris firmis* | J_2_ | Shiti | Xiwan Town, Zhongshan County, Guangxi Province of China | [52] |
| Cydnidae | *Cretacoris gurvanicus* | K_1_ | Gurvan-Eren | West Mongolia | [18] |
|  | *Cydnavites infidis* | K_1_ | Gurvan-Eren | West Mongolia | [18] |
| Mesopentacoridae | *Mesopentacoris* costalis | J_3_ |  | Kazakstan | [66] |
|  | *Mesopentacoris orientalis* | J_2-_ J_3_ |  | West Mongolia | [59] |
|  | *Corienta transbaicalica* | K_1_ | Zaza | eastern Transbaikalia of Siberia | [80] |
|  | *Pauropentacoris macrurata* | K_1_ | Jiufotang | Chifeng City, Inner Mongolia Autonomous Region, China | [81] |

**References**

75. Giebel CG (1856) Fauna der Vorwelt mit steter Berücksichtigung der lebenden Thiere. Bd. 2: Gliederthiere. Abt. 1: Insekten und Spinnen der Vorwelt, mit steter Berücksichtigung der lebenden Insecten und Spinnen, monographisch dargestellt. Leipzig: XVIII. 511pp.

76. Handlirsch A (1925) Palaeontologie. In: Schröder C (eds.) Handbuch der Entomologie, Band 3, Fischer, Jena. pp.117–306.

77. Hong YC (1987) The study of Early Cretaceous fossil insects of Kazuo, west Liaoning. Prof Pap Stratigr Palaeontol 18: 76–85.

78. Hong YC, Wang WL (1990) Fossil insects from the Laiyang Formation. In: The Stratigraphy and Palaeontology of Laiyang Basin Shandong Province. Geological Publishing House, Beijing. pp. 89–105.

79. Popov YuA (1961) A new subfamily of terrestrial bugs in the Jurassic deposits of the Karatau ridge. T Acad Sci USSR Earth Sci Section Paleont 141: 1211–1213.

80．Popov YuA (1990) Description of fossil insects. True bugs. Cimicina. In: Late mesozoic insects of Eastern Transbaikalia. Tr Paleont Inst Acad Sci USSR 239: 20–39.

81. Ren D, Zhu HZ, Lu YQ (1995) New discovery of Early Cretaceous fossil insects from Chifeng City, Inner Mongolia. Acta Geosci Sinica (4): 432–439.
