# Supplementary material for: Phylogeny of the Infraorder Pentatomomorpha Based on Fossil and Extant Morphology, with Description of a New Fossil Family from China
Source: PLoS One. 2012 May 24;7(5):e37289. doi: 10.1371/journal.pone.0037289 (PMC3360028; doi:10.1371/journal.pone.0037289)
Supplement: Text S1 — Character descriptions. (DOC) [file pone.0037289.s004.doc]

**Text S1. Character descriptions.**

[1] General outline of the body: (0) elongate, but length less than 4 times as long as maximum pronotal width; (1) much longer than wide, length at least 6 times maximum pronotal width; (2) ovoid, at most, slightly longer than wide; (3) spheroid, not quite as long as wide.【Among pentatomomorphans, the general outline of the body is often ovoid (e.g. most Pentatomoidea) or elongate (e.g. most Coreoidea, Pyrrhocoroidea and Lygaeoidea). Having a very slender body, (length at least 6 times maximum pronotal width) is an uncommon condition within the Coreoidea, and is only found in some Alydidae Berytidae and Colobathristidae. A spheroid body shape is only found in the Canopidae, Megarididae and Plataspididae.】

[2] Body: (0) not extremely flattened dorsoventrally; (1) extremely flattened dorsoventrally.【In our taxa, the extremely flattened dorsoventrally body shape only occurred in the outgroup, Aradoidea [1, 67].】

[3] Surface of body: (0) smooth, without punctures; (1) sparsely punctuate; (2) densely punctuate. 【The sparsely punctured pronotum and scutellum is the common condition in most of the families of the Pentatomomorpha. Because it is not our purpose to test a detailed scheme of relationships within the Miridae, we have treated all members of that outgroup as having state 0, although we are aware of some members of the Miridae have a punctate pronotum. Members of the Piesmatidae and Malcidae are densely punctate (ie. the diameter of the punctures are as wide as the space between the punctures, usually areolate.】

[4] Shape of head: (0) pentagon; (1) trilateral; (2) head broad, nearly quadrate; (3) semicircular. 【Many families of true bugs exhibit a diversity of head shapes [1, 67].】

[5] Head: (0) conical or sub-conical; (1) dorso-ventrally flattened, laterally carinate. 【This coding was derived from Grazia *et al.* [45], character 2.】

[6] Body foliations (lateral expansions of head, pronotum, corium at base, and abdomen): (0) absent; (1) present. 【This coding was derived from Grazia *et al.* [45], character 3.】

[7] Cephalic trichobothria: (0) present; (1) absent. 【This coding was derived from Schuh *et al.*[36], character 0.】

[8] Spines on lateral margins of head: (0) absent; (1) present. 【Head with lateral spines is only found in the Cydnidae.】

[9] Mandibular plates (juga): (0) short, not extending to apex of clypeus；(1) slightly elongate, extending to apex of clypeus;（2）very enlarged, extending beyond apex of clypeus 【Mandibular plates lengthened, extending to apex of clypeus is found in most Pentatomoidea. The exceptions occur in the families Phloeidae and Urostylididae. Our new fossil has small mandibular plates.】

[10] Bucculae length: (0) long, narrow, extending half the length of head or more; (1) short or reduced. 【This coding is derived from Cassic & Schuh [15], character 19, Henry [11], character 5 and Grazia *et al.* [45], character 11.】

[11] Maxillary plate: (0) absent; (1) present.【This coding is derived from Li [68], character 11 and Li & Zheng [10]. They believed the presence of the maxillary plate is very important evidence supporting the monophyly of the Rhopalidae.】

[12] Length of labium: (0) short, not extending posteriorly to forecoxae; (1) longer, usually extending posteriorly to mesocoxae. 【Labium longer, extending posteriorly to mesocoxae is a relatively common condition in the Pentatomoidea (except Cyrtocoridae, Dinidoridae and Lestoniidae, and the Phyllocephalinae in the Pentatomidae) and all Corioidea (sensu lato).】

[13] Labial segment 1: (0) short; (1) long and relatively slender; (2) virtually absent.【This coding was derived from Schuh *et al.* [36], character6.】

[14] Labium: (0) straight, appressed to body, inserted in ventral surface of head; (1) curved or angular, inserted near anterior surface of head.【This coding was derived from Schuh *et al.* [36], character 5.】

[15] Mandibular and maxillary stylets: (0) normal; (1) extremely elongate and coiled within head. 【maxillary stylets (resting position) coiled within head is found only in the Aradidae and Termitaphididae. 】

[16] Apex of mandibles with (0) row of teeth or pointed transverse ridges; (1) rounded transverse elevations.【This coding was derived from Schuh *et al.* [36], character 8.】

[17] Base of head: (0) forming a ‘neck’; (1) not forming a ‘neck’.【This character and the next character were changed from “character 6” of Grazia *et al.* [45]. We believe the neck length and the distance between the eyes and the pronotum constitute two separate characters.】

[18] Base of head: (0) anterior margin of pronotum distant from eyes; (1) anterior margins of pronotum almost adjacent to eyes.

[19] Post-ocular tubercles: (0) present; (1) absent.【This coding was derived from Grazia *et al.* [45], character 5.】

[20] Shape of eye: (0) round or oval; (1) reniform or kidney-shaped.【Reniform or kidney-shaped eyes are the type typically seen in the Saldidae.】

[21] Compound eyes: (0) rounded, not divided; (1) divided into 2 parts on each side of head; (2) absent.【This coding was derived from Grazia *et al.* [45], character 8.】

[22] Sutures between ocelli and compound eyes: (0) absent; (l) strongly developed.【This coding was derived from Henry [11], character 3.】

[23] Gular groove: (0) absent; (l) present.【This coding was derived from Henry [11], character 4 and Cassis & Schuh [15], character 17.】

[24] Ocelli: (0) present; (1) absent.【Henry coded the Piesmatidae as ocelli absent [11]. We checked some specimens of the Piesmatidae, they actually have ocelli, but they are often hidden under the anterior margin of the pronotomum. We coded Piesmatidae as having ocelli.】

[25] Distance between ocelli and eyes: (0) ocelli closer to eyes than to each other; (1) ocelli closer to each other than to eyes.【This coding was derived from Grazia *et al.* [45], character 10.】

[26] Preocellar pit: (0) absent; (1) present.【This coding was derived from Li [68], character 5 and Li [69], character 5.】

[27] Number of antennal segments: (0) four; (1) five; (2) three. 【This coding was derived from Schuh & Slater [1].】

[28] Antenniferous tubercles: (0) base arising below level of eye, partially obscured by mandibular plates; (1) base arising above level of eye, completely visible in dorsal view; (2) ventral on head, completely covered by mandibular plates.【This coding is derived from Henry [11], character 7, and Grazia *et al.* [45], character 7.】

[29] Antennal segment I: (0) slender, usually longer than head in length; (1) short, stout, barrel shaped, short than half of the head in length; (2) very long, slender, longest of four segments.【A very long first antennal segment occurs only in the Berytidae. In the Mesozoic fossil record, almost all taxa have a short first antennal segment which short than head in length and usually does not attain the apex of the head.】

[30] Shape of antennal segment I: (0) normal, no clubb; (1) conspicuously thickened (clubbed). 【A clavate antennal segment I is found only in the Berytidae.】

[31] Antennal segments in cross section: (0) cylindrical to prismatic, or one preapical segment flattened; (1) at least two preapical segments flattened. 【This coding was derived from Grazia *et al.* [45], character 14.】

[32] Antennal segment 2 length: (0) shorter than segments 3 and 4 combined; (1) very long, distinctly longer than antennal segment 3, subequal to segments 3 and 4 combined; (2) very short, length subequal to diameter.【Antenna 5-segmented and pedicel subdivided are characteristic features of the Pentatomoidea. Corioidea (sensu lato) usually with 4-segmented antenna. Our new fossils with very long pedicel, subequal to segments 3 and 4 combined. We regard pedicel longest and not subdivided as possible evidence that 5-segmented antennae evolved from 4-segmented antennae.】

[33] Antennal segment II: (0) uniformly slender or gradually widened over entire length; (1) clavate apically.【Antennal segment II clavate apically found only in the Berytidae and Malcidae.】

[34] Prepedicellite: (0) absent; (1) present.【This coding was derived from Schuh *et al.* [36], character 11.】

[35] Antennal seglnent IV: (0) slender, not modified or clubbed, similar to segments II and III; (1) clubbed, thickened and fusiform, or globose.【This coding is derived from Henry [11], character 10.】

[36] Pronotum: (0) trapezoidal; (1) hexagon, semicircular or other; (2) anterior margin strong concave, circlelike.【All Mesozoic fossil bugs have trapezoidal pronotum [70], this character is found in almost all Coreoidea, such as the Coreidae, Rhopalidae.】

[37] Pronotum: (0) with a distinct collar; (1) without a distinct collar.【This coding was derived from Li [68], character 12.】

[38] Callus: (0) with distinct callus on pronotum; (1) callus absent.【This coding was derived from Henry [11], character 11 and Cassis & Schuh [15], character22.】

[39] Pronotum: (0) not laterally reflexed; (1) laterally reflexed.【In our taxon, pronotum laterally reflexed is only found in *Pyrrhopeplus carduelis* (Pyrrhocoridae).】

[40] Keel in the middle of the pronotum: (0) absent; (1) present.【This coding was derived from Henry [11], character 13.】

[41] Impressed line on pronotal calli: (0) smooth or punctate, but without transverse impressed line; (1) narrow impressed line or transverse groove present across each callus.【This coding was derived from Henry [11], character 11.】

[42] Lateral margin of pronotum: (0) smoothly rounded; (1) bluntly to sharply carinate.【This coding was derived from Henry [11], character 12.】

[43] Labial groove on thoracic sternum: (0) without sulcus to moderately sulcate; (1) deeply sulcate. 【This coding was derived from Henry [11], character 14, and Cassis & Schuh [15], character 29.】

[44] Carina on thoracic sternum: (0) carina absent or moderately carinate; (1) strongly carinate.【This coding was derived from Henry [11], character 13.】

[45] Metathoracic scent-gland evaporatory structures: (0) evaporatorium and peritreme present; (1) only peritreme present; (2) both evaporatorium and peritreme lacking.【Our coding of this character is often based on references [1, 67, 71], not always on original observations. Openings of the metathoracic scent-gland present is the condition found in all Pentatomomorpha, except in the Pyrrhocoroidea, Rhopalidae and Piesmatidae.】

[46] Peritreme of metathoracic scent gland: (0) never produced; (1) usually produced, often as an elongate spine.【Evaporatorium of metathoracic scent-gland produced, forming an elongate spine is an unique feature of the Berytidae.】

[47] Sent gland: (0) omphalian type; (1) diastomian type.【Sent gland diastomian type is found in Cimicomomorpha and Pentatomomorpha.】

[48] Brindley's gland: (0) absent; (1) present.【This coding was derived from Schuh *et al.*[36], character 18.】

[49] Pronotum: (0) posterior and humeral angles not developed; (1) posterior and humeral angles developed.【This coding was derived from Grazia *et al.* [45], character 15.】

[50] Scutellum: (0) shorter than one-third length of hemelytron; (1) longer than one-third length of hemelytron; (2) absent.【Scutellum longer than than one-third length of hemelytron is found in all Pentatomoidea. The exceptions occur in the Urostylididae, the scutellum triangle and shorter than one-third length of hemelytron. Scutellum absent is only found Termitaphididae.】

[51] Scutellum: (0) not raised basally; (1) raised basally.【Scutellum with elevated base occurs in six families of Pentatomoidea, Aphylidae, Canopidae, Corimelaenidae, Dinidoridae, Plataspididae and Saileriolidae.】

[52] Length of scutellum: (0) short, not or slightly surpassing posterior margin of metathorax; (1) reaching or surpassing an imaginary transverse line crossing the connexivum at apical angles of 3^rd^ abdominal segment; (2) long, almost attaining apex of abdomen but not covering connexivum and corium of hemelytra; (3) well developed, completely covering abdominal dorsum and hemelytra.【This coding was derived from Grazia *et al.* [45], character 16.】

[53] Claval commissure: (0) Clavi overlapping, no claval commissure; (1) well developed; (2) obsolete, claval apices contiguous; (3) absent, claval apices concealed by scutellum; (4) claval apices close together but not contiguous, not concealed by scutellum.【This coding was derived from Grazia *et al.* [45], character 17. Except we added character state (4) as this is the condition found in the new fossils.】

[54] Frena: (0) long, attaining or distinctly surpassing middle of scutellum; (1) short, not surpassing middle of scutellum; (2) obsolete or absent.【This coding was derived from Grazia *et al.* [45], character 8.】

[55] Apical shape of scutellum: (0) simple, unmodified; (1) bifid.【Scutellum apical bifid is the common condition in Malcidae.】

[56] Armature on scutellum: (0) absent; (1) armed with median carinae, conical or globose tubercle, or slender spine.【This coding was derived from Henry [11], character 18.】

[57] Corial margin of forewing: (0) convex or nearly straight, without base of abdomen constricted; (1) narrowed, corial margin concave, with base of abdomen constricted.【Corial margin of forewing convex or nearly straight is the common condition in most of the families of the Pentatomomorpha. In the families Alydidae, Berytidae, Colobathristidae and Malcidae, the forewing is narrowed, the corial margin is concave, with the base of the abdomen constricted.】

[58] Connexivum: (0) completely covered by forewing or scutellum; (1) partially covered by forewing; (2) completely exposed.【The connexivum completely or partially covered by forewing or scutellum is the common condition in most Pentatommomorpha, and is also found in the Miridae and Saldidae. The completely exposed connexivum is only found in Reduviidae, Phloeidae and the new fossils.】

[59] Forewing (0) at most only slightly longer than abdomen, not elbowed at juncture of corium and membrane; (1) much longer than abdomen, elbowed between membrane and corium, and folded below scutellum in repose.【Forewing never elbowed at juncture of corium and membrane is the common condition in most of the families of the Pentatomomorpha. In the Pentatomoidea, three families (Canopidae, Megarididae and Plataspididae) have a much longer forewing, which is elbowed between membrane and corium.】

[60] Forewing: (0) normal; (1) corium very small, separated with claval; (2) absent.【This coding was derived from Schuh & Slater [1].】

[61] Corium: (0) without cell; (1) with one big cells; (2) with more than one cells.【This coding was derived from Schuh & Slater [1].】

[62] Base of corium: (0) not expanded; (1) expanded, foliaceous. 【This coding was derived from Grazia *et al.* [45], character 20.】

[63] Corium：(0) not transparent; (1) at least in part transparent or translucent. 【This coding was derived from Zheng [67]. We follow him in believe in treating the Rhopalidae as having a transparent corium.】

[64] Costal fracture: (0) present, long, delimiting cuneus；(1) present, with corium-membrane boundary subparallel, not delimiting cuneus；(2) absent. 【All extant Pentatomomorpha have the costal fracture absent, but the fossil family, Pachymeridiidae, has a distinctly costal fracture.】

[65] C vein: (0) present；(1) absent. 【In our taxa, only Idiostolidae and Pachymeridiidae have C vein on forewing.】

[66] R, M and Cu veins: (0) inosculated at basal of corium; (1) separate.【R, M and Cu veins inosculated at basal of corium is only found in the new fossils.】

[67] Sc, R, M veins: (0) not diverging at a single point; (1) diverging at a single point. 【Sc, R, M veins diverging at a single point is only found in two families, Idiostolidae and Pachymeridiidae.】

[68] Veins on Clavus：(0) 1A and 2A present; (1) 1A present, 2A absent；(2) both 1A and 2A absent. 【Clavus with 1A and 2A veins is only found in two families, Idiostolidae and Pachymeridiidae. In the new fossils, Reduviidae, and Pyrrhocoridae, only the 1A vein is present.】

[69]Veins on hemelytral membrane: (0) 4 or 5 closed cells; (1) 1 to 3 closed cells; (2) reticulate venation; (3) 6~8 or more simple longitudinal veins; (4) only 3-4 simple longitudinal veins. 【This coding was derived from Schuh *et al.* [36], character 34.】

[70] Distal sector of R+M in hindwing (0) not branching (1) branching. 【Distal sector of R+M in hindwing branching is found in all the Pentatomomorpha and lost in all the outgroups.】

[71] Hind wings: (0) lacking A1 stridulitrum; (1) with A1 stridulitrum. 【This coding was derived from Grazia *et al.* [45], character 22.】

[72] Hamus on hindwing: (0) absent; (1) present.【This coding was derived from Li [68], character 20 and Henry [11], character 19.】

[73] Intercoxal distance: (0) coxae of middle and hind legs more distant from each other than coxae of fore legs; (1) coxae of all three pairs of legs equally distant from each other.【Coxae of all three pairs of legs equally distant from each other is found in most Pentatomomorpha, fossil Rhopalidae, Saldidae and Reduviidae.】

[74] Coxae: (0) glabrous or with a few setae; (1) with fringes of setae, bristles or scales.【This coding was derived from Grazia *et al.* [45], character 26.】

[75] Articulation of metacoxae: (0) directed laterally; (1) directed posterolaterally.【This coding was derived from Henry [11], character 31.】

[76] Distal ends of femora: (0) not conspicuously thickened; (1) conspicuously thickened (clubbed). 【Distal ends of femora conspicuously thickened (clubbed) was found in Berytidae [67] .】

[77] Femora and tibiae of hind legs: (0) normal; (1) frequently incrassate or dilated.【This coding was derived from Zheng [67].】

[78] Femoral trichobothria: (0) absent; (1) present on middle and hind femora.【This coding was derived from Schuh *et al.* [36], character 19.】

[79] Fore tibiae: (0) without a row of stout setae on lateral margin; (1) with a row of stout setae on lateral margin.【This coding was derived from Grazia *et al.* [45], character 27.】

[80] Tibiae sulcate: (0) absent; (1) present. 【This coding was derived from Schuh & Slater [1]. Tibiae sulcate present is only found in Hyocephalidae.】

[81] Fore tibial apparatus: (0) absent; (1) present. 【This coding was derived from Grazia *et al.* [45], character 28.】

[82] Spines on tibiae (0) absent; (1) with 2 or more rows of heavy black or brown spines. 【This coding was derived from Schuh & Slater [1].】

[83] Fossula spongiosa (0) absent; (1) present (at least vestigially). 【This coding was derived from Schuh et al.[36], character 20.】

[84] Number of tarsal segments: (0) three; (1) two.【This coding was derived from Grazia *et al.* [45], character 29, Henry [11], character 33 and Cassis & Schuh [15], character 49.】

[85] Claws (form): (0) cylindrical; (1) flattened, tapering from base to apex.【This coding was derived from Grazia *et al.* [45], character 30.】

[86] Claws: (0) without bristles; (1) with bristles.【This coding was derived from Grazia *et al.* [45], character 31.】

[87] Pretarsus: (0) pulvillus clearly differentiated into basi- and distipulvillus (1) pulvillus obsolete or absent.【This coding was derived from Grazia *et al.* [45], character 32.】

[88] Dorsal arolium: (0) present, elongate, sometimes weakly bladderlike; (1) present, but greatly reduced, forming small bump; (2) absent.【This coding was derived from Grazia *et al.* [45], character 33.】

[89] Dorsal laterotergites: (0) not fused with mediotergites; (1) fused with mediotergites.【This coding was derived from Cassis & Schuh [15], character 56.】

[90] Ventral laterotergites: (0) ventral laterotergites present; (1) not visible and fused with sternum. 【This coding was derived from Cassic & Schuh [15], character 57.】

[91] Abdominal glabrous area: (0) lacking; (1) present.【Abdominal glabrous area was only found in Aradidae.】

[92] Abdominal ventral: (0) lacking disclike organs; (1) with one or two pairs of disc-shaped organs. 【This coding was derived from Grazia *et al.* [45], character 44.】

[93] Abdominal sterna: (0) with a transverse sulcus on each side; (1) without a transverse sulcus on each side.【Megarididae have very special abdominal sterna that have a transverse sulcus on each side.】

[94] Abdominal sternum 3: (0) without a spinelike process; (1) with a long, spinelike, anteriorly projecting process.【Abdominal sternum 3 with a long, spinelike, anteriorly projecting process is only occurs in the Scutelleridae.】

[95] Abdominal pore-bearing organs: (0) lacking; (1) present.【Abdominal pore-bearing organs is only occurs in the Hyocephalidae.】

[96] Sutures of abdominal segment 5: (0) straight; (1) constricted in mid line.【Sutures of abdominal segment 5 constricted in mid line is only found in extant Rhopalidae.】

[97] Connexiva on abdominal segments 5-7: (0) not prominently produced; (1) Connexiva on abdominal segments 5-7 produced into conspicuous dentate lobes.【Connexiva on abdominal segments 5-7 produced into conspicuous dentate lobes is only found in the Malcidae.】

[98] Sternite II at middle: (0) not concealed by metasternum; (1) concealed by metasternum.【This coding was derived from Grazia *et al.* [45], character 38.】

[99] Segmental sutures: (0) all complete; (1) sutures 4 and 5 incomplete. 【This coding was derived from Henry [11], character 24.】

[100] Fusion of abdominal sterna: (0) all segments separate, with conjunctive membranes; (1) sterna 2-4 fused; (2) sterna 2-6 fused; (3) sterna 2-7 fused.【This coding was derived from Henry [11], character 28.】

[101] Connexivum on segment VII: (0) present; (1) absent.【This coding was derived from Henry [11], character 29.】

[102] Position of spiracles: (0) all ventral; (1) only II dorsal, remainder ventral; (2) II, III, and IV dorsal; (3) all dorsal.【This coding was derived from Henry [11], character 23.】

[103] Spiracles on segment VIII in males: (0) spiracles present and not concealed by segment VII; (1) spiracles present but concealed by segment VII; (2) spiracles absent.【This coding was derived from Grazia *et al.* [45], character 39.】

[104] Abdominal trichobothria number per segment: (0) absent; (1) sterna 3-7 with 1-2 trichobothrium on either side of midline; (2) sterna 3-7 with more than 2 trichobothrium on either side of midline. 【This coding was derived from Grazia *et al.* [45], character 34 and Schuh *et al.* [36], character 45.】

[105] Ovipositor: (0) laciniate; (1) platelike; (2) reduced; (3) absent.【This coding was derived from Li 1997, character 70 and Henry [11], character 34.】

[106] Sternite VII: (0) sternite VII split by ovipositor; (1) sternite VII not split by ovipositor, entire. 【This coding was derived from Cassis & Schuh [15], character 59 and Henry [11], character 35.】

[107] Spermatheca: (0) absent; (1) transformed into form of a vermiform gland; (2) present and functional.【This coding was derived from Henry [11], character 36.】

[108] Articulation of paratergite VIII: (0) does not articulate with first valvifer; (1) articulates with first valvifer.【This coding was derived from Henry [11], character 30.】

[109] Tergite IX in females: (0) visible dorsally, not covered by subapically positioned tergite VIII; (1) not visible dorsally, covered by apically positioned tergite VIII; (2) visible ventrally, posterior to segment X.【This coding was derived from Grazia *et al.* [45], character43.】

[110] Eighth paratergite: (0) relatively large, covered ninth paratergite; (1) ninth paratergite visible. 【In our taxa, all of the outgroups and the Idiostoloidea (Idiostolidae+Pachymeridiidae) have relatively small eighth paratergites, leaving the ninth paratergite in view.】

[111] Gonapophyses 8: (0) well developed, first rami distinct; (1) membranous , first rami minute; (2) gonapophyses 8 and first rami lost; (3) gonapophyses 8 fused to gonapophyses 9. 【This coding is derived from Grazia *et al.* [45], character 45.】

[112] Gonapophyses 9: (0) well developed and sclerotized, second rami distinct; (1) moderately sclerotized to membranous, second rami thinly sclerotized or obsolete; (2) reduced, fused to gonocoxites 9, second rami lost.【This coding was derived from Grazia *et al.* [45], character 46.】

[113] Gonangulum: (0) well developed; (1) partially sclerotized or membranous; (2) absent. 【This coding was derived from Grazia *et al.* [45], character 50.】

[114] Ductus receptaculi: (0) dilated or not, but not invaginated; (1) dilated and invaginated, forming three distinct walls, the median one more sclerotized, the vesicular area with the distal aperture open; (2) dilated and invaginated, forming three distinct walls, the median one more sclerotized, the vesicular area with the distal aperture closed. 【This coding was derived from Grazia *et al.* [45], character 51.】

[115] Sternite VIII in males: (0) not or partially covered by segment VII; (1) concealed by segment VII. 【This coding was derived from Grazia *et al.* [45], character 40.】

[116] Tergite VIII in males: (0) sclerotized; (1) membranous. 【This coding was derived from Grazia *et al.* [45], character 41.】

[117] Paramere: (0) variously shaped but never biramous; (1) biramous. 【This coding was derived from Schuh & Slater [1].】

[118] Phallotheca: (0) slightly to moderately sclerotized, relatively flexible; (1) thickly sclerotized. 【This coding was derived from Grazia *et al.* [45], character 55.】

[119] Microchromosome (In-chromosome): (0) absent; (1) present. 【This coding was derived from Leston 1958, Henry [11], character 42, and Zhang & Zheng [72].】

[120] Sex chromosomes: (0) XY; (1) XO. 【This coding was derived from Leston [73], Henry [11], character 42, and Zhang & Zheng [72].】

[121] Number of salivary gland lobes: (0) 1-2; (1) 3; (2) 4. 【This coding was derived from Southwood [74], and Henry [11], character 52.】

[122] Gastric caeca on midgut: (0) absent; (1) present. 【This coding was derived from Grazia *et al.* [45], character 56.】

[123] Dorsal abdominal scent gland between segments 3 / 4 in nymphs: (0) present; (1) absent. 【This coding was derived from Henry [11], character 54 and Schuh & Slater [1].】

[124] Dorsal abdominal scent gland between segments 4 / 5 in nymphs: (0) present (1) absent. 【This coding is derived from Schuh & Slater [1].】

[125] Dorsal abdorninal scent gland between segments 5 / 6 in nymphs: (0) present; (1) absent. 【This coding was derived from Henry [11], character 55 and Schuh & Slater [1].】

[126] Dorsal abdominal scent gland opening in nymphs: (0) single; (1) double, separated; (2) double, separated on raised ‘bumps’. 【This coding was derived from Henry [11], character 56.】

[127] Eclosion fractures: (0) simple slit; (1) hexagonal. 【This coding was derived from Henry [11], character 57.】

[128] Aero-micropylar processes (0) with 1 micropyle; (1) with 2 micropyles；(2) with 3 or more micropyles (3) aeropyles and micropyles combined; 【This coding was derived from Grazia *et al.* [45], character 57.】

[129] Operculum: (0) present; (1) absent. 【Operculum is only occurs in the Aradoidea [1].】

[130] Feeding habits: (0) fungivorous; (1) predaceous; (2) phytophagous. 【Most Pentatomomorpha is phytophagous. Aradidae are usually considered to be fungivorous [1].】

**References**

67. Zheng LY (1999) Class Insecta: Order Hemiptera: Suborder Heteroptera (=Order Hemiptera s. str.). In: Insect Classificatio. Nanjing Normal University Press, Nanjing, China. pp. 442–520.

68. Li XZ (1996) Cladistic analysis and higher classification of Coreoidea (Heteroptera). Entomol Sinica 3(4): 283–292.

69. Li XZ (1997) Cladistic analysis of the phylogenetic relationships among the tribal rank taxa of Coreidae (Hemiptera-Heteroptera: Coreoidea). Acta Zootaxon Sinica 22(1): 60–68.

70. Carpenter FM (1992) Heteroptera. In: Treatise on invertebrate paleontology, Part R, Arthropoda 4, (3). Geological Society of America, Boulder, Colorado, and University of Kansas, Lawrence, Kansas, pp. 259–277.

71. Hsiao TY (1981) A handbook for the determination of the Chinese Hemiptera-Heteroptera. vol. 11, Science Press, Beijing, China. 654pp.

72. Zhang HF, Zheng LY (1998) Chromosomes of Heteroptera: a review of present knowledge. Entomol Know 35(4): 243–246.

73. Leston D (1958) Chromosome number and the systematics of Pentatomomorpha (Hemiptera). Proc Tenth Int Congr Entomol 2: 911–918.

74. Southwood TRE (1955) The morphology of the salivary glands of terrestrial Heteroptera (Ceocorisae) and its bearing on classification. Tijdschr Ent 98: 77–84.
